# The Work and Social Adjustment Scale, Youth and Parent Versions: Psychometric Evaluation of a Brief Measure of Functional Impairment in Young People

**DOI:** 10.1007/s10578-020-00956-z

**Published:** 2020-01-31

**Authors:** Amita Jassi, Fabian Lenhard, Georgina Krebs, Martina Gumpert, Maral Jolstedt, Per Andrén, Martina Nord, Kristina Aspvall, Tove Wahlund, Chloe Volz, David Mataix-Cols

**Affiliations:** 1 National & Specialist OCD, BDD and Related Disorders Clinic for Young People, Maudsley Hospital, London, SE5 8AZ UK; 2 Centre for Psychiatry Research, Department of Clinical Neuroscience, CAP Research Centre, Karolinska Institutet, & Stockholm Health Care Services, Stockholm County Council, Gävlegatan 22, 113 30 Stockholm, Sweden; 3 Institute of Psychiatry, Psychology & Neuroscience, King’s College London, London, UK

**Keywords:** Functional impairment, Disability, Self-report, Parent-report, Psychometric evaluation

## Abstract

The Work and Social Adjustment Scale (WSAS) is a brief global measure of functional impairment that is widely used in adult health. We have adapted the WSAS for its use in youth, the WSAS-Youth version (WSAS-Y) and WSAS-Parent version (WSAS-P). This study evaluated the psychometric properties of the scale. The internal consistency, factor structure, convergent and divergent validity, test–retest reliability and sensitivity to change of the WSAS-Y/P were studied in 525 children and adolescents with obsessive–compulsive disorder and related disorders receiving treatment. The internal consistency of the WSAS-Y/P was excellent across diagnostic groups and time-points. Exploratory factor analysis extracted a single-factor of functional impairment, explaining in excess of 85% of the variance. The test–retest reliability was adequate. The WSAS-Y/P correlated more strongly with other measures of functional impairment than with measures of symptom severity, indicating good convergent/divergent validity. Finally, the WSAS-Y/P was highly sensitive to change after treatment.

**Electronic supplementary material:**

The online version of this article (10.1007/s10578-020-00956-z) contains supplementary material, which is available to authorized users.

## Introduction

The Work and Social Adjustment Scale (WSAS) [1] is a brief (five-item) global measure of functional impairment that is widely used as an outcome measure for adults in clinical psychology/psychiatry. Its psychometric properties have been well established across different psychopathologies and unexplained medical symptoms. Its internal consistency, convergent/divergent validity and test–retest reliability are excellent (e.g. [2, 3]), as are the correlations between the self-report and expert clinicians’ versions of the scale [2, 4]. As an outcome measure, it is highly sensitive to treatment change in a wide range of conditions such as obsessive–compulsive disorder (OCD) [4, 5], bipolar disorder [6], phobic disorders [2], anxiety and depression [7, 8], chronic fatigue syndrome [3], and personality disorder [9, 10].

There is a lack of similar measures specifically designed for young people; existing functional impairment measures include global (i.e. composed of one single scale) and multidimensional (i.e. composed of multiple subscales) instruments. Global measures lack specific information needed for some conditions [11] and are vulnerable to rater bias [12, 13], whereas multidimensional measures often do not differentiate between symptoms and functional impairment [12–14], are time consuming and often require an interviewer [11, 12, 14]. Therefore, there is a need for a pure functional impairment measure that is brief, easy to administer, and that specifies what areas of impairment are to be assessed.

We have adapted the WSAS for its use in youth with a broad range of psychiatric, neurological and functional disorders. The items of WSAS-Youth version (WSAS-Y) are similar to those of the original WSAS but have been adapted to be age-appropriate and to capture areas likely to be functionally affected in young people, such as education. Like the WSAS, the WSAS-Y consists of five items that are rated on a nine-point Likert scale, generating a global score ranging from 0 to 40. The WSAS-Y is accompanied by a parallel parent/guardian version (WSAS-P).

The aim of the current study was to provide a psychometric evaluation of the WSAS-Y/P in a large cohort of youth with OCD and related disorders (including, Body Dysmorphic Disorder, Tourette’s Syndrome and chronic tic disorders, and body-focused repetitive behaviour disorders) treated in specialist child and adolescent mental health services. We hypothesised that, like the original adult version of the WSAS, the WSAS-Y/P would have high internal consistency, a single-factor structure, adequate convergent and divergent validity (that is, we expect significantly higher correlations with other measures of functional impairment than with measures of symptom severity), and test–retest reliability. In addition, we hypothesised that the child and parent versions of the scale would be highly inter-correlated and that both scales would be highly sensitive to change after treatment.

## Method

### Participants

Participants were 525 children and adolescents aged 6 to 19 years with DSM-5 or ICD-10 diagnoses of OCD (*n* = 420), or OCD-related disorders (OCD-RD, *n* = 105) recruited across two European specialist child and adolescent psychiatry centres. The OCD-RD group included individuals with a primary diagnosis of Body Dysmorphic Disorder (BDD, *n* = 33), Tourette’s syndrome or chronic tic disorders (*n* = 55), and body focused repetitive behaviour disorders (trichotillomania or excoriation disorder; *n* = 17).

### Development of the WSAS-Y/P

The WSAS-Y/P was adapted from the original WSAS [1] to make it developmentally appropriate to the functional experiences of young people (see Supplementary Material). Its five items enquire about the extent to which the young person’s current difficulties impair their ability to function in the following areas: school and employment (e.g. summer jobs), everyday activities (e.g. personal hygiene, helping out at home), social activities (e.g. going out with friends), leisure time (e.g. reading, playing videogames) and family/relationships (e.g. parents, siblings, boy/girlfriends). The specific item wording was developed by the authors based on their collective experience working with youth with a range of mental health problems. Preliminary versions were developed and circulated amongst the co-authors for comments and further refinement. Like the original WSAS, for each item, the individual is asked how much their problem (or their child’s problem) impairs their (or their child’s) ability to carry out the activity, with responses ranging from ‘Not at all’ (0) to ‘Severely impaired’ (8). Thus, the WSAS-Y and WSAS-P each generate a total score ranging from 0 to 40, with higher scores indicating higher impairment. The WSAS-Y/P were originally developed in English, then translated into Swedish (by co-author FL) and finally back translated into English by a professional bilingual translator. The senior author (DM-C) supervised the process and accuracy of the Swedish version. Both the English and Swedish versions are available and are free to use for research and healthcare purposes. For permission to translate the scale into other languages, please contact the corresponding author.

### Other Measures

*The Children’s Global Assessment Scale (CGAS)* [15] is a clinician-rated global measure of functional impairment that is associated with the presence of psychopathology. It is scored from 0–100 with higher scores indicating better functioning. The CGAS is reliable between raters and across time and has demonstrated both discriminant and concurrent validity [15].

*Child Obsessive–Compulsive Impact Scale-Revised (COIS-R)* [16] is a 22-item measure of OCD-related functional impairment. There is a parent and child version, both of which demonstrate good internal consistency, concurrent validity, and test–retest reliability [16]. The COIS-R was only administered in the London site to a subgroup of young people (n = 67) with OCD and their parents (n = 68) at baseline.

*The Clinical Global Impressions-Severity (CGI-S)* [17] is a standardized assessment tool that assesses the clinician’s impression of the patient’s current severity of illness. The single item is rated on a seven-point scale, ranging from 1 (normal, not at all ill) to 7 (among the most extremely ill patients) [17, 18]. CGI-S is a widely used measure that has shown good concurrent validity [19] and sensitivity to change [19, 20]. This measure was only used in the Stockholm site.

*The Children’s Yale-Brown Obsessive Compulsive Scale (CY-BOCS)* [21] is a 10-item clinician-rated measure that assesses the severity of OCD symptoms. It is scored between 0 and 40, with higher scores indicating higher severity. The CY-BOCS is a widely used measure in both clinical and research settings, and its psychometric properties are sound [21]. This was administered to the OCD group only (London and Stockholm sites).

### Procedure

The measures used in the present study were collected as part of a larger assessment battery administered to those participating in clinical trials or utilizing clinical services at the Maudsley National and Specialist OCD, BDD and Related Disorders Clinic in London or the Specialist OCD and Related Disorders Clinic in Stockholm. All the individual studies had ethical approval from their respective ethics committees and participants and their parents/guardians provided informed consent/assent.

In all cases, psychiatric diagnoses were made by a multidisciplinary clinical team according to DSM-5 or ICD-10 criteria, typically using semi-structured interviews. All measures, including the WSAS-Y/P, were administered before and after a course of cognitive behaviour therapy (CBT), delivered either face-to-face or via the Internet (ICBT). The mean number of CBT sessions was 13.24 (*SD* = 7.87) and the mean weeks in treatment were 22.20 (*SD* = 23.19).

### Statistical Analyses

We used a classical test theory framework for the psychometric evaluation, including scale reliability, exploratory factor analysis, validity and sensitivity to change. Cronbach’s alpha was used for evaluation of internal consistency of the WSAS-Y/P, and a recommended minimal value of 0.70 was regarded as acceptable [22]. The test–retest reliability of the WSAS-Y/P was examined prior to CBT in two subsets of the sample; one group from the London OCD sample (*n* = 32) that was re-tested 3 weeks post-baseline and one group from the Stockholm OCD sample (*n* = 32) that was retested 12 weeks post-baseline. Exploratory factor analysis with varimax rotation and ordinary least square factor extraction method was conducted to analyse the factor structure of the WSAS-Y/P at baseline and post-treatment, and *R*^2^ is reported as an estimate of the explained variance of the resulting factors. Evaluation of number of relevant factors was made upon visual inspection of scree plots, inclusion of factors with an eigenvalue of ≥ 1 and item factor loadings of ≥ 0.30.

Convergent and divergent validity were tested by pairwise correlations of the WSAS-Y/P and generic measures of functional impairment (e.g. CGAS, COIS) or measures of symptom severity (e.g. CY-BOCS). Stronger correlations between the WSAS-Y/P and other measures of functional impairment (e.g. COIS in OCD patients), than with measures of psychiatric symptoms (e.g. CY-BOCS in OCD patients) would be indicative of adequate convergent/divergent validity.

To evaluate the treatment sensitivity of the WSAS-Y/P, we conducted two paired-sample *t*-tests to calculate the change in WSAS-Y/P scores from pre- to post-treatment. A significant decrease in total score would indicate evidence of sensitivity to change. Furthermore, within-group effect sizes (Cohen’s *d*) were calculated for the WSAS-Y and WSAS-P. Lastly, we conducted correlations of the change scores (post-treatment minus pre-treatment values) of the WSAS-Y and WSAS-P and the CGI-S. A significant correlation of the change scores would further support that the scales are sensitive to change.

## Results

### Sample Characteristics

The age range was 6 to 19 years with a mean age of 14.4 (*SD* = 2.74) and a balanced gender distribution with slightly under half of participants being female. The majority of participants (80%) received traditional face-to-face CBT after the baseline assessment, 14% received ICBT and the remaining 6% received no treatment or were placed on a waitlist condition. The detailed sample characteristics of the groups are shown in Table 1.

**Table 1:** Sample characteristics

	OCD	OCD-RD	Total
N	420	105	525
Age (M, SD)	14.6 (2.64)	13.4 (2.9)	14.4 (2.74)
Sex (% female)	49%	41%	47%
*Treatment (n)*			
Face-to-face CBT	338	82	420
ICBT	48	23	71
None / waitlist	34	–	34
*Site*			
London (n)	164	–	164
Stockholm (n)	256	105	361

*OCD* obsessive–compulsive disorder, *OCD-RD* obsessive–compulsive disorder-related disorders, *CBT* cognitive behaviour therapy, *ICBT* internet CBT

### Internal Consistency

Means and standard deviations of WSAS-Y/P total scores and individual items as well as Cronbach’s alpha values are presented in Table 2. The internal consistency of both versions of the scale was consistently over 0.80, both across diagnostic groups and time points.

**Table 2:** Descriptive statistics and internal consistency of the WSAS-Y/P at baseline and post-treatment across diagnostic groups

	Whole sample	OCD	OCD-RD
*Baseline*			
WSAS-Y (*n*)	415	318	97
Cronbach’s Alpha	.84	.83	.82
Total score (M (SD))	17.17 (10.02)	18.38 (9.88)	13.20 (9.51)
Item 1: school & work (M (SD))	4.13 (2.48)		
Item 2: daily skills (M (SD))	3.49 (2.66)		
Item 3: social activities (M (SD))	3.57 (2.78)		
Item 4: hobbies (M (SD))	2.71 (2.47)		
Item 5: family & relationships (M (SD))	3.36 (2.51)		
WSAS-P (*n*)	424	326	98
Cronbach’s Alpha	.85	.85	.82
Total score (M (SD))	18.81 (10.30)	20.20 (10.23)	14.20 (9.17)
Item 1: school & work (M (SD))	4.69 (2.47)		
Item 2: daily skills (M (SD))	3.77 (2.71)		
Item 3: social activities (M (SD))	4.00 (2.77)		
Item 4: hobbies (M (SD))	2.42 (2.45)		
Item 5: family & relationships (M (SD))	3.92 (2.59)		
*Post-treatment*			
WSAS-Y (*n*)	257	189	68
Cronbach’s Alpha	.89	.90	.82
Total score (M (SD))	9.47 (8.93)	10.25 (9.34)	7.29 (7.30)
Item 1: school & work (M (SD))	2.23 (2.11)		
Item 2: daily skills (M (SD))	1.95 (2.33)		
Item 3: social activities (M (SD))	1.93 (2.17)		
Item 4: hobbies (M (SD))	1.58 (1.95)		
Item 5: family & relationships (M (SD))	1.76 (2.11)		
WSAS-P (*n*)	264	194	70
Cronbach’s Alpha	.88	.89	.84
Total score (M (SD))	10.03 (8.43)	10.59 (8.84)	8.46 (7.02)
Item 1: school & work (M (SD))	2.74 (2.35)		
Item 2: daily skills (M (SD))	2.10 (2.12)		
Item 3: social activities (M (SD))	2.10 (2.18)		
Item 4: hobbies (M (SD))	1.26 (1.66)		
Item 5: family & relationships (M (SD))	1.84 (1.90)		

*OCD* obsessive–compulsive disorder, *OCD-RD* obsessive–compulsive disorder-related disorders, *WSAS-Y* Work and Social Adjustment Scale-Youth Version, *WSAS-P* Work and Social Adjustment Scale-Parent Version

Overall, the OCD group was more severely impaired on the WSAS-Y than the OCD-RD group at baseline (*t* = 4.66, *df* = 164.25, *p* < 0.001), with *M* = 18.38 in the OCD group and *M* = 13.20 in the OCD-RD group. Similarly, there was a significant difference between the groups on the WSAS-P (*t* = 5.5208, *df* = 175.81, *p* < 0.001), with *M* = 20.20 in the OCD group and *M* = 14.20 in the OCD-RD group (Table 2). These differences were mainly driven by the chronic tic and body focused repetitive behaviour disorder groups, which were substantially less impaired at baseline than the OCD and BDD groups (Supplementary Figure).

### Factor Structure

Both the visual scree-plot and the eigenvalue > 1 criteria indicated a one-factor solution for all four exploratory factor analyses (Table 3). The explained variance of the exploratory factor analyses was between 85 and 89%, and all factor loadings were clearly above 0.30.

**Table 3:** Explained variance and factor loadings for the one factor solution of the four exploratory factor analyses of WSAS-Y/P at baseline and post-treatment

	Explained variance/factor loadings	
	WSAS-Y (n = 417)	WSAS-P (n = 425)
*Baseline*		
Explained variance (R^2^)	85%	86%
Item 1: school & work	.71	.71
Item 2: daily skills	.68	.73
Item 3: social activities	.80	.77
Item 4: hobbies	.65	.65
Item 5: family & relationships	.73	.79

	WSAS-Y (n = 257)	WSAS-P (n = 264)
*Post-treatment*		
Explained variance (R^2^)	89%	89%
Item 1: school & work	.79	.82
Item 2: daily skills	.77	.76
Item 3: social activities	.83	.83
Item 4: hobbies	.76	.66
Item 5: family & relationships	.80	.80

*WSAS-Y* work and social adjustment scale-youth version; *WSAS-P*-work and social adjustment scale-parent version

### Test–Retest Reliability

The test–retest Pearson correlations in the London OCD sample (3 weeks retest period) were *r* = 0.80 for the WSAS-Y (*n* = 31), and *r* = 0.57 for the WSAS-P (*n* = 32). In the Stockholm OCD sample (12 weeks retest period) the retest correlations were *r* = 0.69 for the WSAS-Y (*n* = 31) and *r* = 0.81 for the WSAS-P (*n* = 32).

### Convergent and Divergent Validity

Inter-correlations between the WSAS-Y/P and other generic and syndrome-specific measures of general function and symptom severity are presented in Tables 4 and 5. Both versions of the WSAS were highly inter-correlated in both the OCD and OCD-RD groups. In the OCD group (Table 4), the WSAS-Y/P had significantly stronger correlations with other measures of functional impairment (e.g. COIS and CGAS in OCD) than with measures of symptom severity (CY-BOCS in OCD), demonstrating adequate convergent/divergent validity (smallest Z score = 2.6, p = 0.0005). In the OCD-RD group (Table 5), however, the WSAS-Y/P had correlations of similar magnitude with measures of general function (CGAS) and of symptom severity (CGI-S) (largest Z score = -0.4, p > 0.05).

**Table 4:** Scale intercorrelations for the OCD subsample at baseline (total scores, *n* = 420)

	WSAS-P	WSAS-Y	COIS-C	COIS-P	CY-BOCS
WSAS-Y	0.62***				
COIS-C	0.75***	0.78***			
COIS-P	0.77***	0.63***	0.71***		
CY-BOCS	0.31***	0.42***	0.07	− 0.20	
CGAS	− 0.51***	− 0.52***	− 0.45**	− 0.32*	− 0.15*

*WSAS-Y* Work and Social Adjustment Scale-Youth version, *WSAS-P* Work and Social Adjustment Scale-Parent Version, *COIS-C/P* Child Obsessive-Compulsive Impact Scale-Revised-Child/Parent versions, *CY-BOCS* Children´s Yale-Brown Obsessive Compulsive Scale, *C-GAS* Children’s Global Assessment Scale

***p < .001; **p < .01; *p < .05

**Table 5:** Scale intercorrelations for the OCD-RD subsample at baseline (total scores, *n* = 105)

	WSAS-P	WSAS-Y	CGI-S
WSAS-Y	0.72***		
CGI-S	0.60***	0.63***	
CGAS	− 0.67***	− 0.66***	− 0.57***

*WSAS-Y* Work and Social Adjustment Scale-Youth Version, *WSAS-P* Work and Social Adjustment Scale-Parent Version, *CGI-S* Clinical Global Impression-Severity, *C-GAS* Children’s Global Assessment Scale

***p < .001; **p < .01; *p < .05

### Sensitivity to Change

**Fig. 1:**
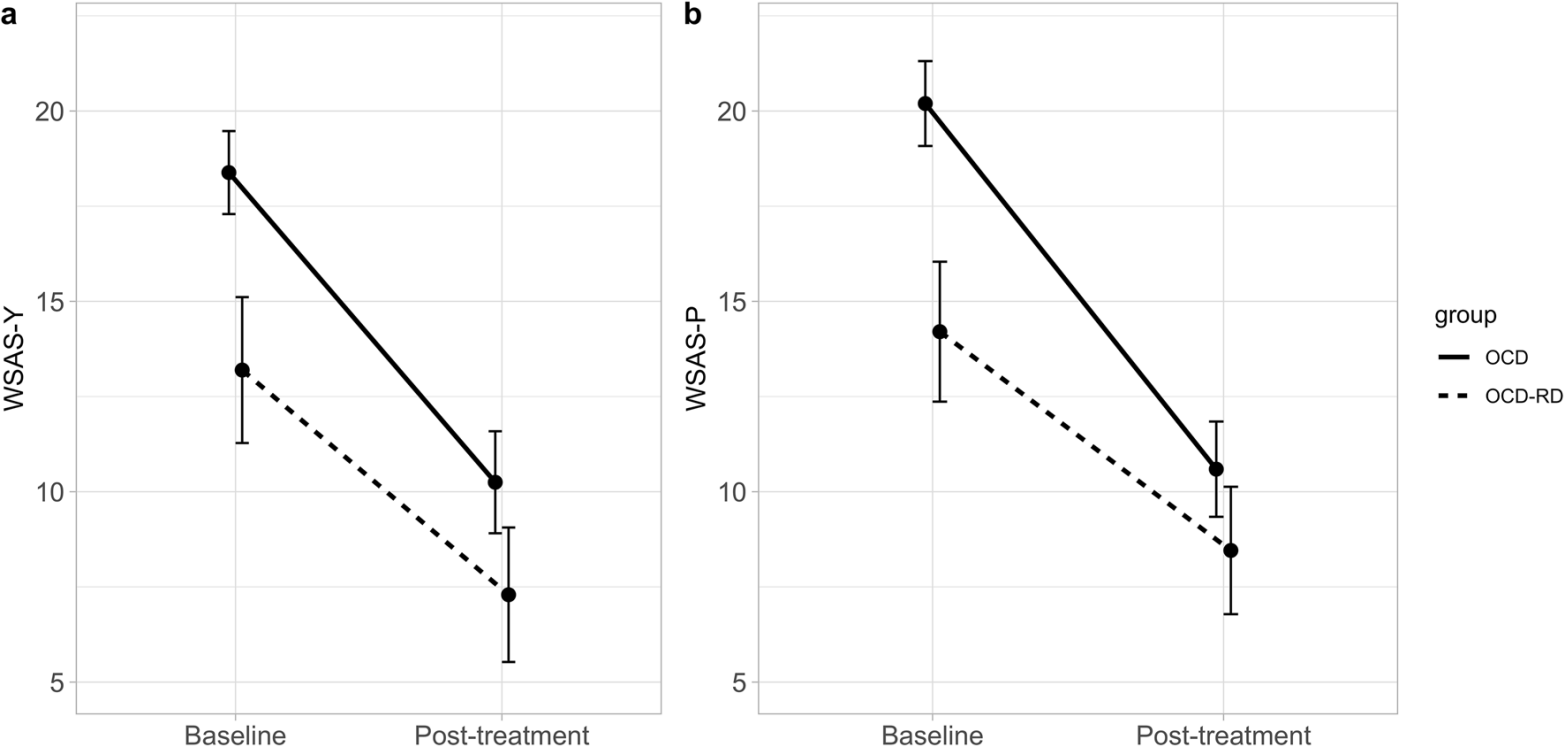
Line graph of baseline to post-treatment means with 95% CIs for WSAS-Y (**a**) and WSAS-P (**b**) by diagnostic group

As shown in Fig. 1, there were significant reductions on the WSAS-Y and WSAS-P from pre- to post-treatment for the combined sample (WSAS-Y; *t* = 11.62, *df* = 373, *p* < 0.001, WSAS-P; *t* = 12.88, *df* = 392, *p* < 0.001), the OCD group (WSAS-Y; *t* = 9.05, *df* = 170, *p* < 0.001, WSAS-P; *t* = 10.56, *df* = 177, *p* < 0.001) and the OCD-RD group (WSAS-Y; *t* = 5.2366, *df* = 65, *p* < 0.001 and for the WSAS-P; *t* = 4.71, *df* = 66, *p* < 0.001). For a graphical representation of symptom reduction in each of the individual disorders in the OCD-RD group, see Supplementary Figure.

For the whole sample, the within-group effect size was Cohen’s *d* = 0.65 (95% CI [0.50, 0.79]) for the WSAS-Y and *d* = 0.80 (95% CI [0.45, 0.75]) for the WSAS-P. The correlation of the WSAS-Y change score with the CGI-S change score was *r* = 0.54 (p < 0.001), and the correlation of the WSAS-P change score with the CGI-S change score was *r* = 0.47 (*p* < 0.001).

## Discussion

This study explored the psychometric properties of the child and parent versions of the WSAS, a widely used measure of functional impairment originally developed for adults, in 525 young people with OCD and OCD-related disorders. In accordance with our first hypothesis, both the child (WSAS-Y) and parent/guardian (WSAS-P) versions had excellent internal consistency across diagnostic groups and time points. Similarly, factor analysis of the scales’ five items consistently extracted a single factor of functional impairment, explaining in excess of 85% of the variance. This was true across versions (child/parent), diagnostic groups (OCD/other) and time points (baseline/post-treatment). Thus, like the adult version of the scale, the WSAS-Y/P measures a unitary construct of functional impairment. While there was some variability across sites and time-frames, the test–retest reliability of the WSAS-Y/P was acceptable. Overall, these results are well in line with a large literature on the psychometric properties of the original, adult version of the WSAS across a range of mental and functional disorders [2–10].

The child and parent versions of the scale (WSAS-Y and WSAS-P) were highly inter-correlated but the correlations were not perfect (0.62 in OCD and 0.72 in OCD-RD), indicating that the two versions of the scale capture partially overlapping constructs. This is a common finding in the clinical assessment of child and adolescent mental health [23]. For this reason, we recommend that both versions be used in tandem. We have not formally evaluated clinician-rated or teacher-rated versions of the scale but these can be easily derived from the youth and parent versions provided herein. Adding such additional versions of the scale would help provide an even more comprehensive picture of the child’s functional impairment across different settings.

We predicted that WSAS-Y/P would display stronger correlations with other measures of functional impairment (e.g. CGAS and COIS in OCD) than with measures of symptom severity (CY-BOCS in OCD). This prediction was confirmed in the OCD sample but not in the OCD-RD sample. This is likely due to the use of a generic symptom severity measure with a limited range of scores (the CGI-S) in the OCD-RD sample, rather than disorder-specific measures, which usually have a wider range of scores. Overall, the results indicate acceptable convergent and divergent validity of the WSAS-Y/P.

Of interest, the OCD group had significantly higher scores on both versions of the scale than the OCD-related group. This difference was mainly driven by the chronic tic and body focused repetitive behaviour disorder groups, who generally had mild symptoms and were well functioning in most areas of life. This indicates that the scale can reliably differentiate between groups known a priori to have different levels of general disability. Future studies should evaluate the psychometric properties of the WSAS-Y/P in additional patient groups (e.g. psychiatric, neurological and functional disorders), and settings (e.g. regular psychiatric clinics, schools).

WSAS-Y/P scores decreased significantly from pre- to post-treatment, indicating good sensitivity to change for each of the diagnostic groups, which is in line with similar work conducted in adult populations [2–10]. The imperfect correlation between the WSAS-Y/P change score and the CGI-S change score (ranging from 0.47 to 0.54) echoes data from multiple clinical trials indicating that CBT is a relatively specific treatment which improves symptoms to a greater extent than it improves other outcomes not directly targeted in treatment, such as functional impairment or quality of life [24].

Limitations of the study include the relatively small number of participants with diagnoses other than OCD, which means that a heterogeneous group of disorders had to be treated as a single group. For the same reason, we had to employ the CGI-S as a generic symptom severity measure, rather than measures specific to each diagnosis. Future studies should expand the number of participants per disorder and include additional conditions. The periods employed to evaluate the test–retest reliability of the scale were different for the London and Stockholm sites (3 and 12 weeks, respectively), which could explain some of the reported inconsistencies between sites.

In conclusion, the WSAS-Y/P is a simple, valid, reliable, and sensitive-to-change measure of functional impairment that retains the sound psychometric properties of the original adult version of the scale.

## Summary

In this paper, we report on the psychometric properties of a brief measure of functional impairment for young people (WSAS-Y and WSAS-P). Tested across two diagnostic groups, the WSAS-Y/P have excellent internal consistency, a single-factor of functional impairment and is strongly correlated with other lengthier measures of functional impairment, indicating good convergent/divergent validity. Finally, the WSAS-Y/P is highly sensitive to change after treatment. This measure has significant scope for use across a range of mental and physical health conditions.

## Electronic supplementary material

Below is the link to the electronic supplementary material.

Supplementary file1 (DOCX 68 kb)

Supplementary file2 (PDF 195 kb)

Supplementary file3 (PDF 136 kb)

## References

[1] Marks IM (1986). Behavioural psychotherapy: Maudsley pocket book of clinical management.

[2] Mataix-Cols D, Cowley AJ, Hankins M, Schneider A, Bachofen M, Kenwright M, Gega L, Cameron R, Marks IM (2005). Reliability and validity of the work and social adjustment scale in phobic disorders. Compr Psychiatry.

[3] Cella M, Sharpe M, Chalder T (2011). Measuring disability in patients with chronic fatigue syndrome: reliability and validity of the work and social adjustment scale. J Psychosom Res.

[4] Mundt JC, Marks IM, Shear MK, Greist JM (2002). The work and social adjustment scale: a simple measure of impairment in functioning. Br J Psychiatry.

[5] Stewart SE, Geller DA, Jenike M, Pauls D, Pauls D, Shaw D, Mullin B, Faraone SV (2004). Long-term outcome of pediatric obsessive-compulsive disorder: a meta-analysis and qualitative review of the literature. Acta Psychiatr Scand.

[6] Fagiolini A, Kupfer DJ, Masalehdan A, Scott JA, Houch PR, Frank E (2005). Functional impairment in the remission phase of bipolar disorder. Bipolar Disord.

[7] Frank E, Cassano GB, Rucci P, Thompson WK, Kraemer HC, Fagiolini A, Maggi L, Kupfer DJ, Shear MK, Houck PR, Calugi S, Forgione RN (2011). Predictors and moderators of time to remission of major depression with interpersonal psychotherapy and SSRI pharmacotherapy. Psychol Med.

[8] Zahra D, Qureshi A, Henley W, Taylor R, Quinn C, Pooler J (2014). The work and social adjustment scale: reliability, sensitivity and value. Int J Psychiatry Clin Pract.

[9] Antonsen BT, Klungsøyr O, Kamps A, Hummelen B, Johansen MS, Pedersen G (2014). Step-down versus outpatient psychotherapeutic treatment for personality disorders: 6-year follow-up of the Ulleval personality project. BMC Psychiatry.

[10] Eikenaes I, Hummelen B, Abrahamsen G, Andrea H, Wilberg T (2013). Personality functioning in patients with avoidant personality disorder and social phobia. J Personal Disorder.

[11] Fabiano GA, Pelham WE, Goldstein S, Naglieri JA (2016). Impairment in children. Assessing impairment: from theory to practice.

[12] Canino G, Costello EJ, Angold A (1999). Assessing functional impairment and social adaptation for child mental health services research: a review of measures. Mental Health Services Res.

[13] Winters NC, Collett BR, Myers KM (2005). Ten-year review of rating scales, vii: scales assessing functional impairment. J Am Acad Child Adolesc Psychiatry.

[14] Bickman L, Lambert EW, Karver M, Andrade AR (1998). Two low-cost measures of child and adolescent functioning for services research. Eval Program Plan.

[15] Shaffer D, Gould MS, Brasic J, Ambrosini P, Fisher P, Bird H, Aluwahlia S (1983). A children's global assessment scale (CGAS). Arch General Psychiatry.

[16] Piacentini J, Peris TS, Bergman RL, Chang S, Jaffer M (2007). Brief report: Functional impairment in childhood OCD: development and psychometrics properties of the child obsessive-compulsive impact scale-revised (COIS-R). J Clin Child Adolesc Psychol.

[17] Guy WBRR (1976). CGI.

[18] Busner J, Targum SD (2007). The clinical global impressions scale: applying a research tool in clinical practice. Psychiatry (Edgmont).

[19] Leon AC, Shear MK, Klerman GL, Portera L, Rosenbaum JF, Goldenberg I (1993). A comparison of symptom determinants of patient and clinician global ratings in patients with panic disorder and depression. J Clin Psychopharmacology.

[20] Khan AE, Broadhead AL, Kolts RL (2004). Relative sensitivity of the Mongomery Åsberg depression rating scale, the Hamilton depression rating scale and the Clinical Global Impressions rating scale in antidepressant clinical trials: a replication analysis. Int Clin Psychopharmacol.

[21] Scahill L, Riddle MA, McSwiggin-Hardin M, Ort SI, King RA, Goodman WK (1997). Children's Yale-Brown obsessive compulsive scale: reliability and validity. J Am Acad Child Adolesc Psychiatry.

[22] Bland JM, Altman DG (1997). Statistics notes: Cronbach's alpha. BMJ.

[23] De Los Reyes A, Augenstein TM, Wang M, Thomas SA, Drabick DA, Burgers DE, Rabinowitz J (2015). The validity of the multi-informant approach to assessing child and adolescent mental health. Psychological Bull.

[24] Carpenter JK, Andrews LA, Witcraft SM, Powers MB, Smits JAJ, Hofmann SG (2018). Cognitive behavioral therapy for anxiety and related disorders: a meta-analysis of randomized placebo-controlled trials. Depress Anxiety..

