# Supplementary material for: The Work and Social Adjustment Scale, Youth and Parent Versions: Psychometric Evaluation of a Brief Measure of Functional Impairment in Young People
Source: Child Psychiatry Hum Dev. 2020 Jan 31;51(3):453–60. doi: 10.1007/s10578-020-00956-z (PMC7235060; doi:10.1007/s10578-020-00956-z)
Supplement: Supplementary file 1 — Supplementary file1 (DOCX 68 kb) [file 10578_2020_956_MOESM1_ESM.docx]

# Supplementary material

**Supplementary Figure**: Line graph of baseline to post-treatment means with 95% CIs for WSAS-Y (a) and WSAS-P (b) by diagnostic group.

## WSAS-Y/P, English version

## WSAS-Y/P, Swedish version

**Supplementary Figure**: Line graph of baseline to post-treatment means with 95% CIs for WSAS-Y (a) and WSAS-P (b) by diagnostic group*.

* T-tests comparing baseline to post-treatment means demonstrated significant mean changes within all diagnostic groups (*p* < .05), apart from the WSAS-P in the body focused repetitive behaviour group (BFRB), which fell just short from significance (*p* =.058).
