# Supplementary material for: The Work and Social Adjustment Scale, Youth and Parent Versions: Psychometric Evaluation of a Brief Measure of Functional Impairment in Young People
Source: Child Psychiatry Hum Dev. 2020 Jan 31;51(3):453–60. doi: 10.1007/s10578-020-00956-z (PMC7235060; doi:10.1007/s10578-020-00956-z)
Supplement: Supplementary file 2 — Supplementary file2 (PDF 195 kb) [file 10578_2020_956_MOESM2_ESM.pdf]

WORK AND SOCIAL ADJUSTMENT SCALE, YOUTH VERSION (WSAS-Y) – Ungdomsversion

Namn: .... Datum: ....

Problem kan ibland påverka ungdomars möjligheter att göra vissa dagliga sysslor eller aktiviteter. För att skatta ditt problem, fundera noga över varje fråga och markera siffran som bäst visar hur mycket ditt problem påverkar dina möjligheter att utföra dessa sysslor eller aktiviteter i din vardag.

SKOLA och ARBETE

Fundera om du kan gå till skolan eller ditt arbete (t.ex. extrajobb) varje dag, om ditt problem gör att du kommer sent eller missar lektioner, och om det påverkar din uppmärksamhet eller hur det går för dig när du pluggar, skriver tentor eller jobbar.

0 ---------- 1 ---------- 2 ---------- 3 ---------- 4 ---------- 5 ---------- 6 ---------- 7 ---------- 8

Mitt problem påverkar det här inte alls.	Mitt problem påverkar det här lite grann.	Mitt problem påverkar det här en del.	Mitt problem påverkar det här mycket.	Mitt problem påverkar det här mycket allvarligt.

VARDAGSSITUATIONER

Fundera på om du kan duscha, bada och klä på dig själv, eller om du har svårt att påbörja eller avsluta sådana saker i tid. Fundera också om du kan hjälpa dina föräldrar med hushållsarbetet eller sysslor i hemmet, ifall de ber om hjälp.

0 ---------- 1 ---------- 2 ---------- 3 ---------- 4 ---------- 5 ---------- 6 ---------- 7 ---------- 8

Mitt problem påverkar det här inte alls.	Mitt problem påverkar det här lite grann.	Mitt problem påverkar det här en del.	Mitt problem påverkar det här mycket.	Mitt problem påverkar det här mycket allvarligt.

SOCIALA AKTIVITETER

Fundera på om du kan träffa dina kompisar och vänner när du vill, till exempel sova över hos en kompis, gå på affärer eller bio med vänner, äta på restaurang, eller att gå på aktiviteter efter skolan.

0 ---------- 1 ---------- 2 ---------- 3 ---------- 4 ---------- 5 ---------- 6 ---------- 7 ---------- 8

Mitt problem påverkar det här inte alls.	Mitt problem påverkar det här lite grann.	Mitt problem påverkar det här en del.	Mitt problem påverkar det här mycket.	Mitt problem påverkar det här mycket allvarligt.

FRITID

Fundera på om du kan göra sådant som du gillar när du är själv, till exempel läsa, titta på tv, spela instrument eller dataspel.

0 ---------- 1 ---------- 2 ---------- 3 ---------- 4 ---------- 5 ---------- 6 ---------- 7 ---------- 8

Mitt problem påverkar det här inte alls.	Mitt problem påverkar det här lite grann.	Mitt problem påverkar det här en del.	Mitt problem påverkar det här mycket.	Mitt problem påverkar det här mycket allvarligt.

FAMILJ och RELATIONER

Fundera på om du kommer överens med de du bor med (dina föräldrar och syskon), eller om du träffar dina vänner eller din pojk-/eller flickvän. Till exempel, kan du åka på semester med familjen, besöka en vän (t.ex. sova över) eller gå på en dejt?

0 ---------- 1 ---------- 2 ---------- 3 ---------- 4 ---------- 5 ---------- 6 ---------- 7 ---------- 8

Mitt problem påverkar det här inte alls.	Mitt problem påverkar det här lite grann.	Mitt problem påverkar det här en del.	Mitt problem påverkar det här mycket.	Mitt problem påverkar det här mycket allvarligt.

WORK AND SOCIAL ADJUSTMENT SCALE, PARENT VERSION (WSAS-P) – Föräldraversion

Namn: .... Datum: ....

Problem kan ibland påverka ungdomars möjligheter att göra vissa dagliga sysslor eller aktiviteter. För att skatta ditt barns problem, fundera noga över varje fråga och markera siffran som bäst visar hur mycket problemet påverkar barnets möjligheter att utföra dessa sysslor eller aktiviteter i sin vardag.

SKOLA och ARBETE

Fundera om ditt barn kan gå till skolan eller sitt arbete (t.ex. extrajobb) varje dag, om problemet gör att han/hon kommer sent eller missar lektioner, och om det påverkar hans/hennes uppmärksamhet eller hur det går för honom/henne när han/hon pluggar, skriver tentor eller jobbar.

0 ---------- 1 ---------- 2 ---------- 3 ---------- 4 ---------- 5 ---------- 6 ---------- 7 ---------- 8

0	2	4	6	8
Problemet påverkar det här inte alls.	Problemet påverkar det här lite grann.	Problemet påverkar det här en del.	Problemet påverkar det här mycket.	Problemet påverkar det här mycket allvarligt.

VARDAGSSITUATIONER

Fundera på om ditt barn kan duscha, bada och klä på sig själv, eller om barnet har svårt att påbörja eller avsluta sådana saker i tid. Fundera också om ditt barn kan hjälpa dig med hushållsarbetet eller sysslor i hemmet, ifall du ber om hjälp.

0 ---------- 1 ---------- 2 ---------- 3 ---------- 4 ---------- 5 ---------- 6 ---------- 7 ---------- 8

0	2	4	6	8
Problemet påverkar det här inte alls.	Problemet påverkar det här lite grann.	Problemet påverkar det här en del.	Problemet påverkar det här mycket.	Problemet påverkar det här mycket allvarligt.

SOCIALA AKTIVITETER

Fundera på om ditt barn kan träffa sina kompisar och vänner när han/hon vill, till exempel sova över hos en kompis, gå på affärer eller bio med vänner, äta på restaurang, eller att gå på aktiviteter efter skolan.

0 ---------- 1 ---------- 2 ---------- 3 ---------- 4 ---------- 5 ---------- 6 ---------- 7 ---------- 8

0	2	4	6	8
Problemet påverkar det här inte alls.	Problemet påverkar det här lite grann.	Problemet påverkar det här en del.	Problemet påverkar det här mycket.	Problemet påverkar det här mycket allvarligt.

FRITID

Fundera på om ditt barn kan göra sådant som han/hon gillar när han/hon är själv, till exempel läsa, titta på tv, spela instrument eller dataspel.

0 ---------- 1 ---------- 2 ---------- 3 ---------- 4 ---------- 5 ---------- 6 ---------- 7 ---------- 8

0	2	4	6	8
Problemet påverkar det här inte alls.	Problemet påverkar det här lite grann.	Problemet påverkar det här en del.	Problemet påverkar det här mycket.	Problemet påverkar det här mycket allvarligt.

FAMILJ och RELATIONER

Fundera på om ditt barn kommer överens med dem han/hon bor med (föräldrar och syskon), eller om han/hon träffar sina vänner eller sin pojk-/eller flickvän. Till exempel, kan barnet åka på semester med familjen, besöka en vän (t.ex. sova över) eller gå på en dejt?

0 ---------- 1 ---------- 2 ---------- 3 ---------- 4 ---------- 5 ---------- 6 ---------- 7 ---------- 8

0	2	4	6	8
Problemet påverkar det här inte alls.	Problemet påverkar det här lite grann.	Problemet påverkar det här en del.	Problemet påverkar det här mycket.	Problemet påverkar det här mycket allvarligt.
