# Supplementary material for: The Work and Social Adjustment Scale, Youth and Parent Versions: Psychometric Evaluation of a Brief Measure of Functional Impairment in Young People
Source: Child Psychiatry Hum Dev. 2020 Jan 31;51(3):453–60. doi: 10.1007/s10578-020-00956-z (PMC7235060; doi:10.1007/s10578-020-00956-z)
Supplement: Supplementary file 3 — Supplementary file3 (PDF 136 kb) [file 10578_2020_956_MOESM3_ESM.pdf]

WORK AND SOCIAL ADJUSTMENT SCALE – YOUTH (WSAS-Y) – Child version

Name: .... Date: ....

Young people's problems sometimes affect their ability to do certain day-to-day tasks in their lives. To rate your problems, think carefully about each item below, and put a circle around the number which best shows how much your problem affects your ability to do these tasks.

SCHOOL/COLLEGE STUDIES and WORK

Try to think about whether you are able to go to school, college or work every day, whether your problem makes you late or miss lessons, and if it affects your concentration or writing when you are trying to study, do exams, or work.

0 ---------- 1 ---------- 2 ---------- 3 ---------- 4 ---------- 5 ---------- 6 ---------- 7 ---------- 8

0	2	4	6	8
My problem doesn't affect this at all.	My problem affects this slightly.	My problem definitely affects this.	My problem affects this a lot.	My problem affects this very severely.

DAILY SKILLS

Try to think about whether you are able to shower, bath, and dress yourself, or if organising your time is difficult. Also consider, if your parents ask, would you be able to help with tasks and chores at home?

0 ---------- 1 ---------- 2 ---------- 3 ---------- 4 ---------- 5 ---------- 6 ---------- 7 ---------- 8

0	2	4	6	8
My problem doesn't affect this at all.	My problem affects this slightly.	My problem definitely affects this.	My problem affects this a lot.	My problem affects this very severely.

SOCIAL ACTIVITIES

Try to think about whether you are able to see your friends when you want to, for example going to sleepovers, going shopping or to the cinema with friends, eating in a restaurant, or going to after-school activities.

0 ---------- 1 ---------- 2 ---------- 3 ---------- 4 ---------- 5 ---------- 6 ---------- 7 ---------- 8

0	2	4	6	8
My problem doesn't affect this at all.	My problem affects this slightly.	My problem definitely affects this.	My problem affects this a lot.	My problem affects this very severely.

HOBBIES

Try to think about whether you can do things that you enjoy when you are by yourself, for example reading, watching TV, playing a musical instrument, and playing computer games.

0 ---------- 1 ---------- 2 ---------- 3 ---------- 4 ---------- 5 ---------- 6 ---------- 7 ---------- 8

0	2	4	6	8
My problem doesn't affect this at all.	My problem affects this slightly.	My problem definitely affects this.	My problem affects this a lot.	My problem affects this very severely.

FAMILY AND RELATIONSHIPS

Try to think about whether you are able to get on with the people you live with (your parents, brother or sister), or spend time your friends or boy/girlfriend. For example can you go on family holidays, stay over at a friend's house, or go on a date?

0 ---------- 1 ---------- 2 ---------- 3 ---------- 4 ---------- 5 ---------- 6 ---------- 7 ---------- 8

0	2	4	6	8
My problem doesn't affect this at all.	My problem affects this slightly.	My problem definitely affects this.	My problem affects this a lot.	My problem affects this very severely.

WORK AND SOCIAL ADJUSTMENT SCALE – YOUTH (WSAS-Y) – Parent version

Name: .... Date: ....

Young people's problems sometimes affect their ability to do certain day-to-day tasks in their lives. To rate your child's problems, think carefully about each item below, and put a circle around the number which best shows how much their problem affects their ability to do these tasks.

SCHOOL/COLLEGE STUDIES and WORK

Try to think about whether your child is able to go to school, college or work every day, whether the problem makes them late or miss lessons, and if it affects their concentration or writing when they are trying to study, do exams, or work.

0 ---------- 1 ---------- 2 ---------- 3 ---------- 4 ---------- 5 ---------- 6 ---------- 7 ---------- 8

0	2	4	6	8
The problem doesn't affect this at all.	The problem affects this slightly.	The problem definitely affects this.	The problem affects this a lot.	The problem affects this very severely.

DAILY SKILLS

Try to think about whether your child is able to shower, bath, and dress without help, or if they find organising time difficult. Would your child be able to help with tasks and chores at home?

0 ---------- 1 ---------- 2 ---------- 3 ---------- 4 ---------- 5 ---------- 6 ---------- 7 ---------- 8

0	2	4	6	8
The problem doesn't affect this at all.	The problem affects this slightly.	The problem definitely affects this.	The problem affects this a lot.	The problem affects this very severely.

SOCIAL ACTIVITIES

Try to think about whether your child is able to see friends when they want to, for example going to sleepovers, going shopping or to the cinema with friends, eating in a restaurant, or going to after school-activities.

0 ---------- 1 ---------- 2 ---------- 3 ---------- 4 ---------- 5 ---------- 6 ---------- 7 ---------- 8

0	2	4	6	8
The problem doesn't affect this at all.	The problem affects this slightly.	The problem definitely affects this.	The problem affects this a lot.	The problem affects this very severely.

HOBBIES

Try to think about whether your child can do things that they enjoy when they are alone, for example reading, watching TV, playing a musical instrument, and playing computer games.

0 ---------- 1 ---------- 2 ---------- 3 ---------- 4 ---------- 5 ---------- 6 ---------- 7 ---------- 8

0	2	4	6	8
The problem doesn't affect this at all.	The problem affects this slightly.	The problem definitely affects this.	The problem affects this a lot.	The problem affects this very severely.

FAMILY AND RELATIONSHIPS

Try to think about whether your child is able to get on with the people they live with (parents, brothers or sisters), or spend time with friends or boy/girlfriend. For example can they go on family holidays, stay over at a friend's house, or go on a date?

0 ---------- 1 ---------- 2 ---------- 3 ---------- 4 ---------- 5 ---------- 6 ---------- 7 ---------- 8

0	2	4	6	8
The problem doesn't affect this at all.	The problem affects this slightly.	The problem definitely affects this.	The problem affects this a lot.	The problem affects this very severely.
